# Effect of gestational diabetes and insulin resistance on offspring’s myocardial relaxation kinetics at three years of age

**DOI:** 10.1371/journal.pone.0207632

**Published:** 2018-11-21

**Authors:** Samuel Blais, Julie Patenaude, Myriam Doyon, Luigi Bouchard, Patrice Perron, Marie-France Hivert, Frederic Dallaire

**Affiliations:** 1 Department of Pediatrics, Université de Sherbrooke, Sherbrooke, Quebec, Canada; 2 Centre de recherche du CHUS, Sherbrooke, Quebec, Canada; 3 Department of Medicine, Université de Sherbrooke, Sherbrooke, Quebec, Canada; 4 Department of Biochemistry, Université de Sherbrooke, Sherbrooke, Quebec, Canada; 5 ECOGENE-21 Biocluster, Chicoutimi, Quebec, Canada; 6 Diabetes Center, Massachusetts General Hospital, Boston, Massachusetts, United States of America; 7 Department of Population Medicine, Harvard Medical School, Harvard Pilgrim Health Care Institute, Boston, Massachusetts, United States of America; Loma Linda University School of Medicine, UNITED STATES

## Abstract

**Purpose:**

Scientific evidence on the long-term impact of gestational diabetes mellitus (GDM) on offspring’s myocardial relaxation is scarce. Studies have linked GDM with transient ventricular hypertrophy in newborns resulting in diastolic dysfunction, but long-term assessment is lacking. The main objective of this study was to evaluate myocardial relaxation in 3-year-old children in relation to the degree of insulin resistance of their mother during pregnancy.

**Methods:**

We prospectively assessed myocardial relaxation by echocardiography imaging on 106 children at 3 years of age. Subjects were divided into 3 groups [GDM, insulin resistance (IR) and normoglycemic (CTRL)], based on their mother’s 75g-OGTT and HOMA-IR results at second trimester screening. We collected information on children adiposity and body size, maternal characteristics and maternal and cord blood measurement of C-peptide and insulin.

**Results:**

The study population comprised 29 children from GDM mothers, 36 children from IR mothers and 41 CTRL children. Compared to the CTRL group, we found that a higher proportion of children in the IR group and the GDM group met the criteria for impaired myocardial relaxation, but this did not reach statistical significance (odds ratio adjusted for heart rate and body surface area of 1.4 [0.2–9.5] and 3.5 [0.6–20.6], respectively).

**Conclusion:**

We did not detect an increased risk of impaired myocardial relaxation at three years of age in children exposed in-utero to IR and GDM, compared to children from normoglycemic mothers.

## Introduction

Abnormal glycemic control during pregnancy and gestational diabetes mellitus (GDM) are frequent, with a prevalence between 6% and 18%, depending on the screening methods.[1] Uncontrolled pre-existing diabetes during pregnancy has been link to several complications in the offspring, including but not limited to fetal macrosomia, shoulder dystocia, neonatal hypoglycemia, neonatal intensive care unit admission, congenital malformations, and stillbirth.[2, 3] Myocardial hypertrophy has often been observed in fetuses and offspring of women with pre-existing and gestational diabetes, and in some cases, even if diabetes was controlled adequately throughout pregnancy.[4–7] The biological mechanisms by which hyperglycemia exposure during fetal growth influences cardiac development are still controversial. Some argue that chronic *in-utero* exposure to hyperglycemia caused by impaired glucose regulation during pregnancy plays a dominant role, while other have suggested that the cause is multifactorial with metabolic, inflammatory and genetic components (reviewed in [3])

Myocardial hypertrophy impairs cardiac muscle relaxation and ventricles’ filling during diastole. Although cardiac hypertrophy in relation to hyperglycemia is usually transient and resolves in the first months of life, some studies have shown that signs of abnormal myocardial relaxation in fetuses and newborns were not always related to hypertrophy and can be present in patients without myocardial hypertrophy.[6, 8, 9] The spontaneous regression of cardiac hypertrophy has led clinicians not to follow these patients after the first few months of life. However, there is a paucity of data on diastolic function in the first years of life in children from mothers with insulin resistance and GDM during pregnancy. To our knowledge, there is no published data on the long-term effect of gestational diabetes and glycemic control during pregnancy on the offspring’s heart function.

We sought to evaluate left ventricular myocardial relaxation in healthy 3-year-old children of women with insulin resistance or diabetes, acquired during pregnancy. Secondary objectives were to evaluate myocardial relaxation in relation to umbilical cord C-peptide and insulin levels. Our hypothesis was that the proportion of children with signs of impaired myocardial relaxation signs is higher in mothers who had higher degree of insulin resistance during pregnancy.

## Materials and methods

### Study population

The study population consisted of offspring of women with gestational diabetes (GDM), women with insulin resistance during pregnancy (IR) and normoglycemic pregnant woman (CTRL) who were selected from the Genetics of Glucose regulation in Gestation and Growth (Gen3G) cohort.[10] The Gen3G cohort was initiated in order to increase the understanding of biological, environmental and genetic determinant of glucose regulation during pregnancy and their impact on fetal and offspring development. The Gen3G cohort enrolled pregnant women without pre-existing diabetes at 1^st^ trimester routine visit, between January 2010 and July 2013 at the Centre Hospitalier Universitaire de Sherbrooke (CHUS). During 2^nd^ trimester medical follow-up, women performed a standard 75g oral glucose tolerance test (OGTT) to determine glycemic status. At delivery, cord blood samples were collected and birth weight was abstracted from electronic medical records. A follow-up of Gen3G mothers and offspring 3 years post-delivery was proposed in order to investigate the consequences of maternal dysglycaemia during pregnancy on offspring. Mothers who accepted to participate in the 3 years follow-up study were also invited to participate to this cardiac function ancillary study. The study was conducted in accordance with the Declaration of Helsinki, the study protocol was approved by the “Comité d’éthique de la recherche en santé chez l’humain du CIUSSS de l’Estrie—CHUS” (approval #: 07-027-A1-M14) and every participant gave informed written consent before enrollment in the study.

We included healthy 3-year-old children free of congenital or acquired cardiac structural anomalies. We excluded children with acute or chronic medical conditions with influence on cardiac output or heart rate (HR): active infection, fever, thyroid problem, arrhythmia, anemia, etc. We also excluded children with 1^st^ degree familial history of cardiomyopathy.

### Categories based on insulin sensitivity indexes

We included children born from women with gestational diabetes (GDM) and gestational insulin resistance (IR), whereas children from normoglycemic women during pregnancy served as controls (CTRL). Gestational diabetes was diagnosed according to the International Association of Diabetes and Pregnancy Study Groups criteria: fasting glucose **>** 5.1 mmol/L, or glucose **>** 10.0 mmol/L 1h-post OGTT, or glucose **>** 8.5 mmol/L 2h-post OGTT.[11, 12] In women whose fasting and post-OGTT glucose levels were comprised within the limits of normal, we used the homeostasis model assessment of insulin resistance (HOMA-IR) to select participants in the IR group and a CTRL group.[13] Children born from women without GDM in the highest quintile of insulin resistance were included in the IR group (HOMA-IR >1.95, equivalent to the 80^th^ percentile of our cohort). To select children born from women without insulin resistance (CTRL group), we targeted women in the lowest quintile of HOMA-IR (<0.79, or below the 20^th^ percentile). To ensure more homogenous groups and increase internal validity, women with HOMA-IR score comprised in the quintiles 2 to 4 where not considered for participation in the study. All participating women were followed-up clinically at our institution. Women with GDM were treated according to national recommendations.[1]

### Data collection

Information about maternal characteristics, pregnancy, and neonatal outcome, including data on GDM diagnosis, results of the OGTT and measures of C-peptide, and insulin were obtained from Gen3G database.[10] At the three-year visit, trained research staff performed standardized anthropometric measures of children. The sum of skin fold thickness measures (average of duplicate measurements of tricep, bicep, subscapular and suprailiac skinfolds) was used as a surrogate measure for adiposity.[10] We calculated the body mass index (BMI) normalized for age and sex according to the World Health Organization standards.[14] Body surface area (BSA) was estimated using the equation proposed by Haycock and collaborators.[15]

### Echocardiography

Two experienced pediatric sonographers performed echocardiography examinations with a Philips iE33 machine (Philips, Eindhoven, Pays-Bas) using a sector probe of 8 MHz. All images were reviewed by a pediatric cardiologist (F. Dallaire) blinded to study groups and poor quality images that precluded accurate assessment of myocardial relaxation were excluded, independently of study groups. We examined children non-sedated while lying on their back or on the left lateral decubitus position. Incidental cardiovascular malformation were searched for and excluded by standard echocardiographic views. We performed all echocardiographic measurements according to the latest recommendation.[16] Measurements were averaged over 3 consecutive beats.

Left ventricle end-diastolic and end-systolic dimensions were acquired in B-mode using the parasternal short-axis view. The following pulse wave Doppler (PWD) measurement were acquired: mitral E wave peak velocity, mitral A wave peak velocity, mitral E wave deceleration time, mitral valve closure to mitral valve opening time, isovolumetric relaxation time, aortic ejection time, left ventricular myocardial performance index (ratio of mitral valve closure to mitral valve opening time minus aortic left ventricular ejection time over aortic left ventricular ejection time), and right upper pulmonary vein diastolic and systolic peak velocities. E’ wave and A’ wave peak velocities of the left ventricle lateral and septal wall were also acquired by tissue Doppler imaging (TDI). All PWD and TDI measurements were normalized for BSA and expressed as Z scores according to previously published reference values.[17]

### Assessment of myocardial relaxation

To limit subjective interpretation of myocardial relaxation, we established cut-off values of PWD and TDI that would correspond, in association, to a pattern compatible with impaired myocardial relaxation: decreased E wave velocity, decreased E/A ratio, increased E wave deceleration time, and decreased TDI lateral E’ wave.[18–20] Hence, myocardial relaxation was considered impaired if the mitral E wave peak velocity was decreased 2.5 standard deviations below the mean (Z score < −2.5) accompanied by at least one other maker of impaired myocardial relaxation: decreased E/A peak velocity ratio > 1 standard deviation below the mean (Z score < −1.0), increased E wave deceleration time > 1 standard deviation above the mean (Z score >1.0), or decreased TDI lateral e’ wave peak > 1 standard deviation below the mean (Z score < −1.0).

### Laboratory measurements

Insulin and C-peptide cord-blood levels were used as a surrogate measure of glycemic control during pregnancy as suggested by the HAPO study.[11] Maternal blood at 2^nd^ trimester and cord blood were stored at –80°C until analysis. Insulin and C-peptide concentrations were quantified in maternal blood at 2^nd^ trimester and in cord blood using multiplexed particle-based flow cytometric assays (Luminex technology, Millipore Corp, Billerica, MA, USA). Maternal plasma glucose was quantified using the glucose hexokinase method (Roche Diagnostics, Indianapolis, IN, USA). Intra- and inter-assays coefficients of variation are 1.0% and <1.7% for glucose and < 10% and <15% for C-peptide and insulin, respectively.

### Statistical analyses

We used SAS for Windows version 9.4 for all analyses (SAS Institute Inc., Cary, NC, USA). Data are presented as median and interquartile range. Most variables were not normally distributed. Therefore, medians were compared using the non-parametric Mann–Whitney U test. We used logistic regression to estimate the odds ratio of impaired myocardial relaxation according to study groups adjusting for resting HR and BSA. A p-value < 0.05 was considered statistically significant.

## Results

### Population and demographics

Out of the 1024 women enrolled at 1^st^ trimester of pregnancy in the Gen3G cohort, 695 could be contacted and 448 accepted that their offspring be included in the three-year follow-up. Of them, 187 met inclusion criteria for this ancillary study and were invited to participate. One hundred eleven subjects accepted to take part in the study. Four children were excluded because of lack of cooperation and/or inadequate acoustic windows, and one was excluded because of an incidental finding of a bicuspid aortic valve. A total of 106 children were included in the final analysis.

Participant’s characteristics are presented in Table 1. HOMA-IR was significantly different in GDM and IR groups compared to controls, as would be expected (1.68 vs 0.64, p<0.01 and 2.49 vs 0.64, p<0.01, respectively). Median pregravid BMI were higher in IR group compared to the CTRL group (26.4 vs. 21.8, p<0.01). Median concentration of cord blood C-peptide was higher in the IR group compared to the control group (181 pmol/L vs 139 pmol/L, p = 0.02). Pregravid BMI and cord blood C-peptide were also slightly higher in the GDM group but differences did not reach statistical significance. Compared to children in the CTRL group, children in the GDM group had higher BMI-for-age Z-score (0.98 vs. 0.23, p = 0.02) and higher total skinfold thickness (31.0 mm vs. 27.8 mm, p = 0.01) at 3 years. Children in the IR group also had slightly higher BMI-for-age Z-score and higher total skinfold thickness at 3 years compared to the CTRL but the differences did not reach statistical significance.

**Table 1:** Study population characteristics.

	All participants n = 106 Median (IQR)	Controls n = 41 Median (IQR)	Insulin resistance n = 36 Median (IQR)	Gestational diabetes n = 29 Median (IQR)	IR vs CTRL (p-value)	GDM vs CTRL (p-value)
**Mothers**						
Age at delivery (years)	28.0 (5.0)	28.5 (4.0)	28.00 (6.0)	29.00 (7.0)	0.47	0.79
Pregravid BMI (kg/m^2^)	23.6 (8.4)	21.8 (3.6)	26.40 (10.2)	23.90 (6.9)	< 0.01	0.08
HOMA-IR	1.67 (1.81)	0.64 (0.24)	2.49 (0.97)	1.68 (1.65)	< 0.01	< 0.01
C-peptide in cord blood (pmol/L)	161 (107)	139 (106)	181 (185)	171 (77)	0.02	0.11
Insulin in cord blood (mUI/L)	9.35 (5.47)	9.07 (5.31)	9.88 (9.08)	9.54 (6.77)	0.33	0.63
**Children**						
Sex (% males)	43.4	36.6	44.4	51.7	0.48	0.21
Birth weight (g)	3460.0 (475.0)	3390.0 (370.0)	3510.0 (584.0)	3435.0 (640.0)	0.16	0.84
Age at echocardiography (years)	3.35 (0.29)	3.36 (0.32)	3.35 (0.28)	3.35 (0.31)	0.71	0.64
Weight at echocardiography (kg)	15.13 (2.70)	14.75 (2.45)	15.35 (2.35)	15.05 (2.80)	0.64	0.84
Height at echocardiography (cm)	97.05 (5.20)	97.80 (4.60)	96.79 (5.48)	96.50 (4.10)	0.55	0.26
Body surface area at echocardiography (m^2^)	0.64 (0.07)	0.64 (0.06)	0.65 (0.06)	0.64 (0.08)	0.65	0.92
BMI-for-age Z score at echocardiography	0.44 (1.22)	0.23 (1.17)	0.38 (0.78)	0.98 (1.38)	0.26	0.03
Sum of skin folds at echocardiography (mm)	29.50 (10.00)	27.75 (7.50)	31.25 (11.25)	31.00 (7.00)	0.07	0.01
Heart rate at echocardiography (bpm)	97.00 (15.00)	96.00 (14.00)	99.00 (16.00)	98.00 (16.00)	0.45	0.46

### Echocardiography measurements

Table 2 shows echocardiography absolute measurements and Z score according to groups. Overall, the median of all parameters did not differ between groups. We observed a median mitral peak E-wave velocity somewhat lower than previously reported reference values (median Z score of –1.17) but this was constant across groups. LV mass was comparable between groups and no patient had evidence of hypertrophy subjectively, or abnormal LV mass according to reference values by Foster et al.[21]

**Table 2:** Echocardiography measurements.

	All participants n = 106 Median (IQR)	Controls n = 41 Median (IQR)	Insulin resistance n = 36 Median (IQR)	Gestational diabetes n = 29 Median (IQR)	IR vs CTRL (p-value)	GDM vs CTRL (p-value)
Estimated left ventricular mass (g)	27.79 (6.86)	28.18 (7.60)	26.85 (6.38)	28.07 (6.30)	0.20	0.45
Mitral E wave peak velocity (cm/s)	95.8 (17.6)	95.8 (13.0)	96.3 (19.1)	95.1 (18.1)	0.52	0.89
Mitral E wave peak velocity (Z score)	-1.17 (1.31)	-1.15 (1.04)	-1.14 (1.52)	-1.23 (1.35)	0.54	0.97
Mitral A wave peak velocity (cm/s)	50.8 (10.4)	50.0 (8.5)	52.3 (11.6)	50.7 (12.4)	0.39	0.24
Mitral A wave peak velocity (Z score)	-0.61 (0.87)	-0.72 (0.80)	-0.49 (0.89)	-0.66 (1.07)	0.30	0.27
Mitral E wave deceleration time (seconds)	136.1 (33.0)	136.3 (31.7)	135.0 (44.8)	136.0 (24.3)	0.93	0.82
Mitral E wave deceleration time (Z score)	0.55 (1.58)	0.57 (1.48)	0.44 (2.15)	0.58 (1.16)	0.88	0.81
E/A ratio	1.92 (0.54)	1.93 (0.52)	1.96 (0.65)	1.83 (0.43)	0.32	0.36
E/A ratio (Z score)	-0.08 (1.13)	-0.04 (0.98)	-0.01 (1.37)	-0.31 (0.89)	0.35	0.32
TDI lateral E’ wave peak velocity (Z score)	-0.30 (1.02)	-0.25 (0.81)	-0.36 (1.00)	-0.30 (1.31)	0.11	0.40
E/E’ ratio	5.81 (1.17)	5.67 (0.74)	5.88 (1.27)	6.03 (1.44)	0.38	0.40

### Risk of impaired myocardial relaxation

Signs of impaired myocardial relaxation were observed in 11/106 subjects (10.4%). These include one subject with normal mitral inflow velocities but abnormal septal and lateral TDI e’ wave velocities, abnormal e’/a’ wave ratio, and abnormal E/e’ ratio that was considered to have abnormal relaxation with pseudo-normalization. Table 3 shows the proportion of children with impaired myocardial relaxation in each group as well as odds ratio (OR) for IR and GDM groups compared to CTRL group. Compared to the CTRL, signs of impaired myocardial relaxation was more likely in the IR group [adjusted OD 1.4 (95% confidence interval 0.2–9.6)] and in the GDM group [adjusted OD 3.5 (95% confidence interval 0.6–20.0)], although the differences did not reach statistical significance.

**Table 3:** myocardial relaxation according to study groups.

	Proportion (%)			Odds ratio [95% CI]*	
	Control	RI	DBG	RI versus CTRL	DBG versus CTRL
Participants with abnormal relaxation	2/41(4.9%)	4/36 (11.1%)	5/29(17.2%)	Unadjusted OR 2.4 [0.4–14.2] Adjusted OR 1.4 [0.2–9.5]	Unadjusted OR 4.1 [0.7–22.6] Adjusted OR 3.5 [0.6–20.6]

OR = odds ratio.

*OR were adjusted for resting heart rate and body surface area.

When cord blood C-peptide and insulin levels were examined in relation with myocardial relaxation, we found higher median cord blood C-peptide and insulin levels in subjects with impaired myocardial relaxation, but this did not reach statistical significance. Median level of cord blood C-peptide levels were 185 pmol/L (IQR of 127) in children with impaired myocardial relaxation compared to 155 pmol/L (IQR of 97) in those without (p = 0.23). Median cord blood level of insulin levels were 10.75 mUI/L (IQR of 5.13) in children with impaired myocardial relaxation compared to 9.08 mUI/L (IQR of 5.53) in those without (p = 0.38).

We explored whether resting HR could explain the relatively high proportion of children with impaired myocardial relaxation. Resting HR was significantly correlated with mitral E-wave peak velocity and mitral E/A ratio (Table 4). There was also a weak correlation between resting HR and mitral E-wave deceleration time (Table 4). Consequently, children with higher HR where more likely to be classified as having impaired myocardial relaxation. Median HR in children meeting criteria for impaired myocardial relaxation was higher compared to children not meeting criteria for impaired myocardial relaxation (106 bpm (IQR of 14) vs. 97 bpm (IQR of 14), p = 0.02).

**Table 4:** Correlation between HR and echocardiographic measurements of ventricular relaxation.

Echocardiography measure	Pearson correlation coefficients with resting heart rate (p-value)
Mitral E wave peak velocity Z score	-0.207 (p = 0.03)
E/A ratio Z score	-0.380 (p<0.01)
Mitral E wave deceleration time Z score	-0.189 (p = 0.06)
TDI lateral E’ wave peak velocity Z score	-0.165 (p = 0.09)

## Discussion

In this study, we found a relatively high proportion of children meeting our criteria of impaired myocardial relaxation. Higher proportion of children in the IR group and the GDM group met criteria for impaired myocardial relaxation compared to controls, but this did not reach statistical significance. Children meeting criteria for impaired myocardial relaxation also had higher resting HR. Despite re-analysis, we found no way of disentangling the effect of HR from that of impaired myocardial relaxation, as both affect PWD velocities. It is thus difficult to conclude if IR or GDM during pregnancy had a lasting effect on the myocardial relaxation in offspring at 3 years of age.

This study underlines the difficulty in identifying long-term effect of intra-uterine environment on heart function. If changes in myocardial architecture do occur *in-utero* as a consequence of exposure to a hyperglycemic environment or other pathophysiologic processes related to maternal insulin resistance, we did not detect obvious changes in diastolic function indices at 3 years of age. Further studies are needed to assess if there is indeed a higher risk of impaired myocardial relaxation associated with insulin resistance and gestational diabetes, and if this translates into increased long-term cardiovascular risk.

Two recent studies compared ventricular function at 30 weeks gestation in approximately 50 fetuses of normoglycemic women to fetuses exposed to hyperglycemia (both pre-gestational and gestational diabetes during pregnancy). The results of these studies showed differences in PWD velocities or differences in myocardial deformation that could be compatible with impaired myocardial relaxation in fetuses exposed to a hyperglycemic uterine environment.[8, 9] In both studies, authors observed increased septal thickness in fetuses from diabetic women. However, Miranda et al. showed that differences in myocardial deformation were independent of septal hypertrophy.[9]

In 1991, Mehta et al. had already identified altered diastolic filling at 2–4 weeks in infant from pre-gestational diabetic women, which had resolved at 6–9 weeks.[22] More recently, Zablah et al. used TDI to show what they called a “subclinical decrease in systolic and diastolic myocardial function” in newborns from GDM women compared to normoglycemic women during pregnancy.[23] Similar findings were reported in 2015 by Al-Biltagi et al.[4] We could not find any published data on the effect long-term effect of gestational diabetes or pre-existing type 2 diabetes during pregnancy on the offspring. In 7- to 8-year-old children born to women with type I diabetes, Rijpert et al. did not find any difference in cardiac dimensions or function compared to controls.[24]

Most PWD and TDI indices are influenced by body size, preload and HR in young children. In designing this study, we assumed that it would be difficult to identify differences across groups by simply comparing the mean values of each of these indices. We thus approached this by establishing a set of criteria of body size-adjusted PWD and TDI indices that, together, would be compatible with reduced myocardial relaxation. Our first criterion was a clearly decreased mitral E-wave peak velocity (Z < –2.5). However, our sample had a median mitral E-wave peak velocity lower that published reference values, which may have lowered our threshold of abnormal myocardial relaxation. Nevertheless, we decided not to change our initial hypothesis *a posteriori* based on this observation.

This study has limitations. The number of children included in each group is limited and our study may have been underpowered. The effect of HR, as mentioned above, may affect most PWD and TDI indices. It is unclear if the difference of HR across group is due to chance or related to myocardial relaxation. Furthermore, the observed effect of hyperglycemia on diastolic function may have been blunted by the treatment administered to GDM women after diagnosis was established. This may also explain the lack of significant difference between insulin and C-peptide level when comparing GDM and control groups. We did not have echocardiography examination at birth and we cannot relate our results with the presence of myocardial hypertrophy or diastolic function impairment at birth. Children in the DBG group had higher BMI-for-age, which could lead to underestimation of Z scores when Z score equations are based on BSA.[25] To correct for this effect, we adjusted our model for BSA. At this age, it is unclear if being overweight could affect myocardial relaxation. Including BMI-for age in the model did not change the results.

## Conclusion

In this study, we did not detect significant differences in offspring exposed to IR or GDM during in utero development and children from normoglycemic women during pregnancy regarding the probability of meeting criteria of impaired myocardial relaxation at three years of age. There was a non-statistically significant increased risk of impaired myocardial relaxation in children in the IR group and the GDM group, but it remains unclear if the higher resting HR in these groups has played a role. Further studies are needed to conclude if IR or GDM during pregnancy has a lasting effect on myocardial relaxation in offspring.
